# Thermal reference points as an index for monitoring body temperature in marine mammals

**DOI:** 10.1186/s13104-015-1383-6

**Published:** 2015-09-04

**Authors:** Mar Melero, Víctor Rodríguez-Prieto, Ana Rubio-García, Daniel García-Párraga, José Manuel Sánchez-Vizcaíno

**Affiliations:** VISAVET Center, Veterinary School, Complutense University of Madrid, Avenida Puerta de Hierro, s/n., 28040 Madrid, Spain; Veterinary Services, Oceanografic, Parques Reunidos Valencia, Ciudad de las Artes y las Ciencias, 46013 Valencia, Spain

**Keywords:** Thermography, Thermal pattern, Blowhole temperature, Eye temperature, Cetaceans, Pinnipeds

## Abstract

**Background:**

Monitoring body temperature is essential in veterinary care as minor variations may indicate dysfunction. Rectal temperature is widely used as a proxy for body temperature, but measuring it requires special equipment, training or restraining, and it potentially stresses animals. Infrared thermography is an alternative that reduces handling stress, is safer for technicians and works well for untrained animals. This study analysed thermal reference points in five marine mammal species: bottlenose dolphin (*Tursiops truncatus*); beluga whale (*Delphinapterus leucas*); Patagonian sea lion (*Otaria flavescens*); harbour seal (*Phoca vitulina*); and Pacific walrus (*Odobenus rosmarus divergens*).

**Results:**

The thermogram analysis revealed that the internal blowhole mucosa temperature is the most reliable indicator of body temperature in cetaceans. The temperatures taken during voluntary breathing with a camera held perpendicularly were practically identical to the rectal temperature in bottlenose dolphins and were only 1 °C lower than the rectal temperature in beluga whales. In pinnipeds, eye temperature appears the best parameter for temperature control. In these animals, the average times required for temperatures to stabilise after hauling out, and the average steady-state temperature values, differed according to species: Patagonian sea lions, 10 min, 31.13 °C; harbour seals, 10 min, 32.27 °C; Pacific walruses, 5 min, 29.93 °C.

**Conclusions:**

The best thermographic and most stable reference points for monitoring body temperature in marine mammals are open blowhole in cetaceans and eyes in pinnipeds.

## Findings

### Background

Marine mammals have adapted to the aquatic environment after a long evolution process that started 60 million years ago [1]. Since they live partially or entirely in water, thermoregulation is a significant challenge. Entirely aquatic mammals, like cetaceans, must constantly maintain a body temperature within narrow limits in an environment where heat is conducted 25 times faster than in air [2, 3]. Amphibious mammals like pinnipeds require additional mechanisms to maintain the physiological temperature range when they leave water. Since slight variations in body temperature may indicate body dysfunction [4], infection [5] or physiological processes like estrus and proximity to parturition [6], accurate measurements and easy body temperature monitoring are very useful in veterinary care.

Since body temperature could be measured directly in marine mammals only after death [7], radio-pills were developed which, when ingested, allow body temperature measurements in live animals [8]. However, this method is costly, affected by food ingestion, some individuals may vomit the device, and reader has to remain reasonably close to the animal to record the temperature. At present, the body temperature in live marine mammals is usually estimated from rectal readings [6, 9, 10]. Rectal temperature closely correlates with body temperature [11, 12]. However, measuring rectal temperature in marine mammals requires special probes, training or restraining, can induce stress in animals, and requires time to obtain measurements, which mean that it is unsuitable under certain circumstances; e.g., newborns, untrained animals, or animals in the wild. Moreover, the depth at which the rectal probe enters influences the temperature reading as testes (located on both sides next to the rectum) and uterus are cooled by the vascular countercurrent heat exchanger [13–16]. In certain circumstances, taking rectal temperature might even require anaesthetising the animal because some species and individuals do not tolerate rectal temperature measurements [5], including domestic animals like dogs as only 68 % tolerate it [17]. In addition, physiological processes like digestion or peristalsis, the presence of faecal masses, and the level of muscle tone and physical activity may alter rectal temperature measurements [18].

New technologies have been developed to avoid the disadvantages of these temperature measurement methods. One is infrared thermography, in which thermal radiation at wavelengths of between 0.8 and 1.0 mm is collected and quantified from the animal to be transformed into a digital image [4]. This technique offers many advantages. It can be used at a long distance from the animal [19], it reduces handling stress and ensures technicians’ safety. It can generate thermograms of many individuals in a short period of time and serves as a non-invasive diagnostic tool since it can detect variations in surface body temperature due to trauma, inflammation, infection, neurological processes, neoplasm, heat emission from internal organs or heating by external sources [10].

Infrared thermography has been applied to study many domestic species [20] and wildlife [21], including marine mammals [22–25]. Eye temperatures measured by thermography have been shown to be practically the same, or at least correlate with body temperatures in several mammal species [5, 26, 27], including human beings [28]. However, reference points on the surface of an animal that can be used reliably to estimate body temperature have not been established for most species, especially those studied mainly in the wild. This manuscript aims to apply thermography to five species of marine mammals in order to establish thermal reference points as an index of body temperature based on its easy measurement and its variance.

## Methods

Thermographic measurements were carried out in two cetacean species, namely bottlenose dolphin (*Tursiops truncatus*) and beluga whale (*Delphinapterus leucas*). Three pinniped species were also studied: Patagonian sea lion (*Otaria flavescens*), harbour seal (*Phoca vitulina*), and Pacific walrus (*Odobenus rosmarus divergens*). The study was performed in December 2007 and April 2008 with the assistance of animal trainers.

All the animals were housed at the Oceanografic Aquarium of Valencia, and all the national and international permits were in order. No special permits were required for this research because thermograms were taken without modifying the animals’ behaviour and rectal temperature measurements were performed within the Oceanografic’s routine procedures.

The air and water temperature facilities were monitored twice daily. Bottlenose dolphins, harbour seals and Patagonian sea lions were housed in independent outdoor facilities where the air temperature ranged between 8.2 and 22.0 °C in December 2007 and between 18.0 and 20.0 °C in April 2008. Water temperature was maintained between 16.6 and 16.8 °C for bottlenose dolphins, between 17.6 and 18.1 °C for Patagonian sea lions and between 16.1 and 16.7 °C for harbour seals. Belugas and walruses were housed in indoor facilities located in the same building. The air temperature there was maintained between 15 and 18 °C, and the water temperature was kept between 13.5 and 15.0 °C for belugas and between 14.6 and 15.8 °C for walruses.

For cetaceans, eye and blowhole thermograms were obtained from a distance of less than 2 m while the animal was breathing voluntarily and on demand in the water. Ten adult bottlenose dolphins (two males and eight females) and two adult beluga whales (one male and one female) were imaged. During the sessions, which lasted no more than 22 and 15 min, 253 and 284 thermograms of eyes and 67 and 74 of blowholes were taken in dolphins and belugas, respectively.

For pinnipeds, the thermograms of ears, eyes, nostrils, vibrissal area, mouth and axillas were obtained. Eleven adult Patagonian sea lions (two males and nine females) and two male pups were imaged. During the sessions, which lasted no more than 18 min, 243 thermograms were taken as soon as the animals left the pool, with the camera positioned at less than 5 m from the animals. Six adult harbour seals (three males and three females) and one female cub were imaged. During the sessions, which lasted no more than 25 min, 264 thermograms were taken from a distance of less than 10 m and as soon as the animals poked their heads out of the water. Five juvenile Pacific walruses (one male and four females) were imaged. During the sessions, which lasted no more than 65 min, 388 thermograms were taken after the animals left the pool, with the camera positioned less than 5 m from the animal.

A ThermaCAM E45 Infrared Camera with a FOV25 lens (FLIR, Burlington, ON, Canada) was used for the thermographic measurements. Its thermal sensitivity is 0.1 at 25 °C. Images were analysed by the ThermaCAM QuickReport 1.0 software (FLIR), and the maximum temperatures in each area of the thermograms were calculated. Since blowholes remain open only briefly during breathing, the thermographic videos were recorded. Later in order to ensure the most accurate measurement, each video frame was analysed individually to calculate the maximum temperature inside the blowhole. Since video frames could not be evaluated with this software, only in this case, temperature measurements correspond to a little bigger area than the anatomical region evaluated. A significant relationship between dorsal fin surface temperature and water temperature have been previously determined, being the animal surface around 0.9 °C warmer than the water [25]. In this study, as expected, all temperature measurements of the surrounding skin were cooler than the interior of the blowhole, so the maximum temperature value always correspond to a point located inside it. In order to evaluate the stabilisation period of eye temperatures, thermograms were taken every 30 s after the animal left the water.

The emissivity of these animals was assumed to be 0.96, the same as water [29], and as Cuyler et al. [24] established for marine mammals given the thin film of water that covers their body surface, the tear film on their eyes or normal secretion in respiratory mucosa. The angle between the camera and the animal was taken into account because of its relevance in measurement accuracy [24, 25]. Hence, the values were divided into two groups: perpendicular, i.e., when the camera was situated perpendicularly to the animal; inclined, i.e., at angles over 30º.

In all the cetaceans, the rectal temperatures were measured at the same time as the eye and blowhole temperatures using a flexible probe thermometer with a Propaq Encore 202EL monitor (Welch Allyn, Skaneateles Falls, NY, USA). In order to perform temperature measures in a region anterior to the countercurrent heat exchanger, rectal probe was positioned at 40 cm deep to the anus in bottlenose dolphin, as Rommel et al. [14] described; and at 50–60 cm in beluga whale. To ensure temperature values were not influenced by the countercurrent heat exchanger, it was verified during the retreat of the probe that higher temperatures were not registered. Rectal temperatures in pinnipeds were measured only in three walruses and in two sea lions because the remaining animals were not trained for this purpose.

For the statistical analysis, the maximum temperatures and standard deviations of each area were calculated and the Pearson product-moment approach was followed to evaluate the correlation between the rectal temperature and the simultaneous eye and blowhole measures with the SAS 9.1.3 software (SAS Institute, Cary, NY, USA). A Kruskal–Wallis test was done to compare the temperature of each anatomical area according to the age and gender of the animals, and the hour of the day; for this purpose, the software R, version 2.15.2, was employed (The R Foundation for Statistical Computing, 2012). For all the comparisons, a value of *p* < 0.05 was considered statistically significant.

## Results and discussion

Temperatures values were obtained for all the animals for almost all the thermographic areas studied, and the means, standard deviations and correlations were calculated (Table 1).

**Table 1:** Temperature values of marine mammals measured by thermography

	Patagonian sea lion	Harbour seal	Pacific walrus	Bottlenose dolphin	Beluga whale
Rectal	37.07 ± 0.058	–	36.22 ± 0.383	36.75 ± 0.369	35.68 ± 0.184
Ears	–	22.81 ± 6.030	–		
Eyes					
Overall	10 min 31.13 ± 1.706 0.873*	10 min 32.27 ± 0.901	5 min 29.93 ± 1.015 0.599*	1 min 32.93 ± 1.053 0.801*	1 min 32.14 ± 0.847 0.607*
Males	31.38 ± 1.518	32.89 ± 0.352	=	=	x
Females	29.56 ± 1.448	31.49 ± 0.756	=	=	x
Pups	22.29 ± 2.344	21.73 ± 1.972	x	x	x
Adults	28.55 ± 2.885	27.07 ± 5.434	x	x	x
Vibrissal area					
Overall	26.23 ± 4.220	23.54 ± 6.764	23.75 ± 3.739		
Males	27.37 ± 3.641	29.91 ± 4.779	=		
Females	24.29 ± 2.778	18.04 ± 1.757	=		
Pups	18.94 ± 1.323	18.11 ± 1.445	x		
Adults	26.20 ± 3.649	25.32 ± 6.879	x		
Mouth	–	–	30.89 ± 3.191		
Blowhole					
Voluntary					
Perpendicular				36.86 ± 0.297 0.997*	34.73 ± 0.665 0.994*
Inclined				33.5 ± 2.246	33.21 ± 1.537
On demand					
Perpendicular				33.92 ± 1.256 0.448*	31.82 ± 0.670 0.876*
Inclined				30.06 ± 2.098	28.76 ± 1.687

Maximum temperatures (mean ± standard deviation) of each analysed anatomical region of the five species of marine mammals, displayed in Celsius degrees (°C). [Patagonian sea lion (*Otaria flavescens*), harbour seal (*Phoca vitulina*), Pacific walrus (*Odobenus rosmarus divergens*), bottlenose dolphin (*Tursiops truncatus*), beluga whale (*Delphinapterus leucas*)]

–: Not enough valuable thermal measurements were collected

=: Differences were found not to be significant using a Kruskal–Wallis test (*p* < 0.05)

x: Not enough individuals of different gender or age were studied to estimate these parameters

* Correlation with rectal temperature

**Fig. 1:**
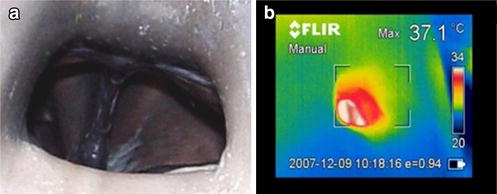
Blowhole of a bottlenose dolphin (*Tursiops truncatus*). Digital (**a**) and thermographic (**b**) images of the blowhole during voluntary breathing with the thermal camera placed perpendicularly to the longitudinal axis of the dolphin

For cetaceans, the thermographs of eyes and blowholes were analysed (Table 1). Eye temperature stabilised equally rapidly (within 1 min), and a steady-state value was obtained (Table 1). The stabilisation time was shorter than in pinnipeds, probably because the anatomical and physiological differences of marine mammals’ eyes as cetaceans have well-developed vascular multivessel plexuses [30, 31]. Blowhole temperature accuracy depended on where the thermal camera was positioned in relation to the animal, and also depending on whether or not the thermal data were collected during voluntary breathing or breathing on demand. The lowest standard deviation was reached at the mean maximum blowhole temperature during voluntary breathing, when thermograms were taken perpendicularly to the surface of the blowhole opening (Fig. 1). Variation in the blowhole temperature was even greater according to whether or not animals were breathing voluntarily or on demand, irrespectively of whether animals were inspiring or exhaling (Table 1).

The mean rectal temperature was 36.75 ± 0.369 °C (mean ± standard deviation) in bottlenose dolphins and 35.68 ± 0.184 °C in beluga whales. The blowhole gave the highest correlation with rectal temperature when measured perpendicularly during voluntarily breathing (Table 1). The poorest correlation during breathing on demand was probably due to the shorter time that the blowhole remained open and due to its smaller aperture, which limited measurement quality.

**Fig. 2:**
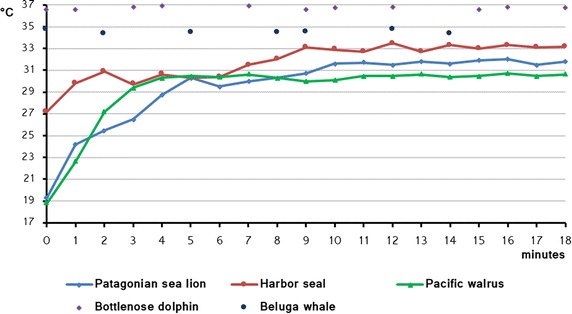
Pinnipeds eye temperature stabilisation and cetaceans blowhole temperature along time. Pinnipeds eye temperature for 18 min after leaving the water of one individual of each different species under study [Patagonian sea lion (*Otaria flavescens*), harbour seal (*Phoca vitulina*), Pacific walrus (*Odobenus rosmarus divergens*)]. Cetaceans blowhole temperature during voluntary breathings measured perpendicularly to the longitudinal axis of one individual of each evaluated specie [Bottlenose dolphin (*Tursiops truncatus*), beluga whale (*Delphinapterus leucas*)]

Our results suggest that, in cetaceans, blowhole is superior to eyes as a thermal reference point during voluntary breathing, it obtain the lowest variance and the highest correlation with rectal temperature, and a temperature stabilisation period is not needed (Fig. 2). When thermograms were taken perpendicularly during voluntary breathing, the temperature was practically the same as the rectal temperature in bottlenose dolphins and was only 1 °C lower than the rectal temperature in beluga whales (Table 1).

This suggests that the thermography of blowhole opening can be a useful tool for monitoring body temperature in captive cetaceans as it is more rapid and less invasive than measuring rectal temperature. In individuals with an injury or a local inflammatory process that affects the blowhole area or upper airways, eyes can be an alternative index for body temperature.

For pinnipeds, six anatomical areas were analysed by thermography. We considered that eyes were the best thermal reference point in these animals because eye temperature gave the lowest standard deviation (Table 1), obtained a stable value, was easy to measure and no training was needed. Eye temperature increased gradually after leaving the water to then stabilise after a period of time depending on the species (Fig. 2). Similarly, the steady-state temperature achieved depended on the species (Table 1). Some variations of eye temperature, lower than 0.8 °C, were registered after the stabilisation period. These differences were considered small, since Morgan et al. [32] established a difference of 0.6 °C between both eyes of the same individual as the upper limit for 95 % of the normal human population. The mean rectal temperature was 37.07 ± 0.058 °C in Patagonian sea lions and 36.22 ± 0.383 °C in Pacific walruses. Eyes gave the highest correlation with rectal temperature when measured after the temperature stabilisation period. However, as it was not possible to measure rectal temperature in harbour seals, we were unable to directly correlate eye temperature with body temperature.

For the remaining anatomical regions, we were unable to collect enough valuable measurements from nostrils and axillas to do a statistical analysis. Likewise, ear measurements and mouth values were considered only for harbour seals and Pacific walruses, respectively (Table 1). This situation came about given the difficulty to access these anatomical areas, requirement of training to maintain the correct position, or temperatures continued to be similar to the rest of the body, so it was not possible to assess statistical differences. The thermography of the vibrissal area was highly variable; this region is often affected by the appearance of physiological thermal windows, which make accurate measurements very difficult (Table 1).

The Kruskal–Wallis test revealed statistically significant differences according to gender in Patagonian sea lions (eyes and vibrissal area temperatures), harbour seals (ears, eyes and vibrissal area temperatures) and bottlenose dolphins (blowhole during voluntary breathing). Likewise, significant differences were found in accordance with the age of Patagonian sea lions (eyes and vibrissal area temperatures) and harbour seals (ears, eyes and vibrissal area temperatures). According to these parameters, males obtained higher temperature values than females, and adults presented higher temperatures than pups. However, as all the individuals belonged to the same group, it was not possible to evaluate the effect of gender in belugas and the effect of age in walruses, dolphins and belugas. No significant differences in the reference temperatures after the stabilisation periods were found in accordance with circadian rhythm, and the air and water temperatures for any of the species examined. The robustness of this method should be tested with a larger number of animals, and with a broader range of physiologic, pathologic and ambient conditions, particularly in natural habitats.

Further studies are required to establish the equivalence between rectal and eye or blowhole temperature in a larger number of individuals, in hyper- and hypothermic animals, and also under a wider range of environmental conditions.

## Conclusions

In cetaceans, the best thermographic reference point for body temperature is the blowhole, and the thermal data should be collected when the animal is breathing voluntarily with the camera held perpendicularly to its blowhole opening. Under these conditions, the mean maximum blowhole temperature is practically the same as the rectal temperature in bottlenose dolphins and is only 1 °C lower in beluga whales. In pinnipeds, the eye can be a good thermographic reference point as an index for monitoring body temperature, but measurements should be taken only after a stabilisation period, which is characteristic for each species.

These results suggest that thermography is a reliable, immediate, remote, non-invasive tool that can prove useful for taking thermal measurements of the anatomical referent points to monitor body temperature in marine mammals. This technique is especially useful for untrained animals because previous training is not necessary, it is safe for technicians and it avoids stressing the animals.

## Abbreviations

°C: Degree celsius
min: Minutes
m: Metre
cm: Centimetre
mm: Millimetre
µm: Micrometre
